# Insecticidal activity of two proteases against *Spodoptera frugiperda *larvae infected with recombinant baculoviruses

**DOI:** 10.1186/1743-422X-7-143

**Published:** 2010-06-29

**Authors:** Aline Welzel Gramkow, Simone Perecmanis, Raul Lima Barbosa Sousa, Eliane Ferreira Noronha, Carlos Roberto Felix, Tatsuya Nagata, Bergmann Morais Ribeiro

**Affiliations:** 1 Cell Biology Department, University of Brasília, Brasília, DF, CEP 70910-970, Brazil

## Abstract

**Background:**

Baculovirus comprise the largest group of insect viruses most studied worldwide, mainly because they efficiently kill agricutural insect pests. In this study, two recombinant baculoviruses containing the ScathL gene from *Sarcophaga peregrina *(vSynScathL), and the Keratinase gene from the fungus *Aspergillus fumigatus *(vSynKerat), were constructed. and their insecticidal properties analysed against *Spodoptera frugiperda *larvae.

**Results:**

Bioassays of third-instar and neonate *S. frugiperda *larvae with vSynScathL and vSynKerat showed a decrease in the time needed to kill the infected insects when compared to the wild type virus. We have also shown that both recombinants were able to increase phenoloxidase activity in the hemolymph of *S. frugiperda *larvae. The expression of proteases in infected larvae resulted in destruction of internal tissues late in infection, which could be the reason for the increased viral speed of kill.

**Conclusions:**

Baculoviruses and their recombinant forms constitute viable alternatives to chemical insecticides. Recombinant baculoviruses containing protease genes can be added to the list of engineered baculoviruses with great potential to be used in integrated pest management programs.

## Background

Baculovirus comprise the largest group of insect viruses most studied worldwide, mainly because they efficiently kill agricultural insect pests. They are specific to one or a few related insect species [1], and have infectious particles protected in protein crystals which allows the formulation of biopesticides with easy application technology. Their use as boinsectides are a safe alternative to chemical insecticides [2,3].

They are large double-stranded, circular DNA viruses with a genome size ranging from 80 to 200 kilobases (kb) [4]. Baculoviruses have enveloped rod-shaped virions and two distinct phenotypes in a single cycle of infection: the budded virus (BV), which is responsible for transmitting the virus from cell to cell and the occlusion-derived virus (ODV), which is occluded in a proteinaceus occlusion body, [5] and is responsible for horizontal transmission of the virus from insect to insect.

The type species of the Baculoviridae family is the *Autographa californica multiple nucleopolyhedrovirus *(AcMNPV) which is the most studied baculovirus at the molecular level, having a wide spectrum of hosts and has been widely used as an expression vector for heterologous proteins in insect cells and insects [6]. To speed up the death of their hosts, recombinant baculoviruses have been constructed, increasing their biopesticide properties. Some of the most effective recombinant baculoviruses are the ones containing insect-specific neurotoxins genes [7-9]. In susceptible hosts, these neurotoxins, expressed during virus infection, reduce damage to crops and decrease the time required to kill the insects from 25 to 50% when compared to larvae infected with the wild type virus [10-14].

Besides insect-specific toxins, other proteins have been introduced into the genome of baculoviruses. For instance, one of the first effective recombinant baculovirus constructed with the intention of improving biological control, contained the diuretic hormone gene from *Manduca sexta *that, when injected into larvae of *Bombyx mori*, was able to kill the insects 20% faster than wild-type virus [15]. The wild type and mutant juvenile hormone esterase (JHE) genes from *Heliothis virescens *were also inserted into the genome of AcMNPV [16-19]. The wild type JHE gene has shown an improvement on AcMNPV pathogenicity only towards *Trichoplusia ni *neonate larva [16]. However, mutated versions of the JHE gene that improved protein stability also showed increased pathogenicity towards *H. virescens *larvae [20]. Some baculoviruses produce during infection, the enzyme Ecdysteroid UDP-Glycosyl Transferase (EGT), which inactivates the hormone ecdysone of their hosts [21,22]. The deletion or inactivation of the *egt *gene can also results in reduced infected-insect time to death and reduced economic damage to crops [21,23].

Recombinant baculoviruses have also been constructed with enhancin genes from other baculoviruses. These recombinants were based on AcMNPV and were designed to improve the ability of the virus to gain access to midgut epithelium cells [24-26]. Also chitinases of some insects pathogens have also been used to increase baculovirus pathogenicity [27,28]. Some entomopathogenic microbes produce chitinases to penetrate the insect host body [27,29] and baculoviruses themselves also produce chitinases to liquefy the host body after their death by viral infection [30,31]. Another type of toxin gene used with the purpose of increasing baculovirus pathogenicity is the Cry toxin gene from *Bacillus thuringiensis *(Bt). Some Cry toxin genes were inserted into AcMNPV genome and shown to produce large amounts of biological active toxins [32-37]. However, only a Cry toxin fused with the major occlusion body protein (polyhedrin) of the baculovirus AcMNPV was capable of improving the virus pathogencity towards its insect host [37].

The only protease gene used with the aim of improving insecticidal activity of baculoviruses was the cathepsin-L (ScathL) gene of *Sarcophaga peregrina*, which showed reduced survival time and damage caused by infected larvae when compared with the wild virus [38].

*Spodoptera frugiperda *(Lepidoptera: Noctuidae) is a polyphagous species that attacks many economically important crops in several countries. In Brazil, this insect can attack the following crops: corn, sorghum, rice, wheat, alfalfa, beans, peanuts, tomato, cotton, potatoes, cabbage, spinach, pumpkin and cabbage [39,40].

*Aspergillus fumigatus *is found in nature as an opportunistic pathogen of the airways, affecting humans, birds and other animals. It is responsible for a variety of respiratory diseases and many invasive infections. This fungus produces many proteolytic enzymes such as elastases [41-43], serine proteases [44] and collagenases [45], which are involved in many key events in the pathophysiology of *A. fumigatus *[46]. The Keratinase of the fungus *A. fumigatus *has been isolated, purified and characterized previously [46].

In this study, we constructed recombinant baculoviruses containing the ScathL gene from *S. peregrina*, and the Keratinase gene from the fungus *A. fumigatus*, under the command of two promoters in tanden and analysed their insecticidal properties against *S. frugiperda *larvae.

## Methods

### Virus and cell

*Trichoplusia ni *insect cells (BTI-Tn5B1-4) [47] and/or *S. frugiperda *IPLB-Sf21-AE (Sf-21) [48] were kept at 27°C in TC-100 medium supplemented with 10% fetal bovine serum (GIBCO-BRL). These cell lines were used for the *in vitro *propagation of AcMNPV and the recombinant vSynVI-gal, which contains the β-galactosidase (*lac-Z*) gene in place of the *polh *gene [49], and were also used for the construction of the recombinant viruses containing the ScathL and Keratinase genes, respectively.

### Construction of recombinant plasmids and viruses

The cathepsin-L (ScathL) gene from *S. peregrina *was amplified by PCR using specific oligonucleotides (Protease F 5'-CCACCAGCAACCATCACCTT*AAGCTT*TAACAC-3') (Protease R 5'-*GAATTC*AATTGAAAAAGGCAG-3') and DNA from the pKYH5 plasmid (courtesy of Dr. Robert Harrison, Iowa State University, USA). The Protease F oligonucleotide anneals at positions -10 to -35 and relative to the start codon (ATG) and the Protease R oligonucleotide anneals to positions +76 to +91 relative to the last nucleotide of the stop codon (TAA) of the ScathL gene. The position of the *Hin*dIII and *Eco*RI restriction sites are shwon in italics, respectively. The amplified fragment was then cloned into vector pGEM^®^-T following the manufacturer's instructions (Promega). The plasmid pGEMScathL containing the gene for ScathL was digested with *Nco*I (Invitrogen) and *Not*I (Promega), the resulting fragment was separated by electrophoresis in an agarose gel (0.8%) and the fragment of 1,100 bp, corresponding to the ScathL gene was purified using the DNA extraction Perfect Gel Cleanup kit, according to manufacturer's instructions (Eppendorf). Next, we carried out a T4 DNA polymerase reaction (Invitrogen) using the purified fragment in order to create blunt ends, following the manufacturer's instructions (Invitrogen) and ligated the fragment to the transfer vector pSynXIVVI+X3 [49], which enables insertion of the heterologous gene under the control of two promoters in tandem (pSyn and pXIV) [49], and previously digested with *Sma*I and dephosphorylated, according to the manufacturer's protocol (Promega). *Escherichia. coli *DH5α cells were transformed with the ligation by electroporation [50] and the recombinant plasmid (pSynScathL) was obtained. The plasmid pGEMKerat containing the Keratinase gene from *A. fumigatus *[46] was amplified in DH5α cells of *E. coli *and purified using the DNA extraction Concert kit, according to manufacturer's instructions (Invitrogen). The plasmid was digested with *Eco*RI (GE), the DNA fragment corresponding to 1,200 bp was purified from an agarose gel (0.8%), using the GFX DNA extraction kit, according to the manufacturer's instructions (GE). The purified fragment was ligated with the *Eco*RI-digested and dephosphorilated transfer vector pSynXIVVI+X3 [49], using the Rapid DNA Ligation^® ^kit following the manufacturer's instructions (Promega). The ligation product was then used to transform DH5α cells in order to obtain the transfer vector pSynKerat.

The plasmid DNAs from pSynScathL and pSynKerat (1 μg each) were separately co-transfected with the DNA (0.5 μg) of the *Bsu*36I-linearized vSynVI-gal recombinant virus in BTI-TN-5B1-4 cells (10^6^), using liposomes following the manufacturer's instructions (CellFectin^®^, Invitrogen ).

Seven days after co-transfection, the supernatants of the co-transfected cells were collected and used for the isolation of the recombinant viruses vSynKerat and vSynScathL by serial dilution in 96-well plates [51].

### Bioassays

Thirty 3^rd ^instar *S. frugiperda *larvae (for each virus) were injected with 10 μl of each viral stock (approximately 1 × 10^6 ^pfu) into the hemolymph, as a negative control, thirty *S. frugiperda *larvae were also injected with culture medium and the experiment was repeated three times. The inoculated larvae were placed individually in plastic cups with artificial diet and observed twice daily until death. Statistical analysis was performed using the Polo Plus program (LeOra Software).

Bioassays with occluded viruses were conducted using the droplet feeding method [52] with five different concentrations of occlusion bodies per nanoliter (10^2^, 10^1^, 1.0, 0.1, 0.01 occlusion bodies/nL). Thirty neonate larvae of *S. frugiperda *were used for oral inoculation with the different viral doses from each of the recombinant viruses, the wild type AcMNPV and with only dye (2% phenol red) as negative control. Mortality was scored until 10 d.p.i. and the data analyzed by probit analysis using the Polo Plus program (LeOra Software). The insects were monitored every eight hours for ten days. The inoculated larvae were placed individually in plastic cups with artificial diet and the experiment was repeated three times. The mean time to death (TD) was calculated according to Morales et al. [53].

### Structural and ultrastructural analysis of the internal tissues of virus-infected *S. frugiperda *larvae

Ten 3^rd ^instar *S. frugiperda *larvae were injected with the recombinant viruses as described above and dissected at different times post infection. The insects were dissected by cutting along their backs with an entomological scissors to expose the gut and other organs and were photographed under a stereomicroscope (Stemi SV 11, Zeiss). An uninfected larvae was used as control. Furthermore, the infected insects were also prepared for scanning electron microscopy, as described in Matos et al. [54]. Briefly, the infected insects were fixed in a solution of 2% glutaraldehyde and 2% paraformaldehyde in sodium cacodylate buffer 0.1 M, pH 6.4 for 2 h at 4°C, washed by 3 cycles of 15 min with cacodylate buffer 0.1 M and post-fixed in osmium tetroxide and 1:1 potassium ferrocyanide for 2 h and then dehydrated with an ascending series of acetone and then dried (Balzer CPD30 critical point drier) and covered with gold in an sputter coater apparatus (Balzer SCD 050). The samples were then analyzed in a scanning electron microscope JEOL JSM 840 at10 kV.

### Phenoloxidase activity

Third-instar *S. frugiperda *larvae were separately inoculated with BV stocks (10^8 ^pfu/mL) with AcMNPV, vSynScathL, vSynKerat and mock infected as described above. At 72 h p.i., haemolymph was collected and placed into 100 μl of anticoagulant buffer (0.098 M NaOH, 0.186 M NaCl, 0.017 M EDTA, 0.041 M Citric acid) and used for detection of phenoloxidase activity. Briefly, hemolymph samples were kept on ice, and hemocytes were pelleted by centrifugation at 3,000 × *g *for 5 min at 4°C. The cell-free hemolymph, 113 μg, was then transferred to a tube containing 800 μL of 10 mM L-3,4-dihydroxyphenylalanine (L-DOPA) and incubated for 20 min at 25°C and the mixture analyzed in a spectrophotometer at 475 nm.

## Results

### Construction of recombinant plasmids and viruses

**Figure 1:**
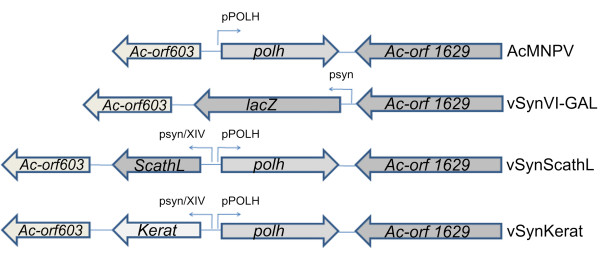
**Scheme showing the polyhedrin loci of AcMNPV wild type and different recombinant baculoviruses**. The *polh *(polyhedrin), *lac-Z *(β-galactosidase), *ScathL *(cathepsin) and *Kerat *(Keratinase), *Ac-orf603 *and *Ac-orf1629 *genes are shown in the figure. The position of the pSyn/XIV and pPOLH promoters are also shown.

The ScathL gene from *S. peregrina *was amplified by PCR from pKYH5 plasmid DNA and cloned into the vector pGEM^®^-T Easy (data not shown). The DNA fragment containing the gene was removed from the cloning vector by digestion with restriction enzymes and cloned into the transfer vector pSynXIVVI+X3 forming a new plasmid, called pSynScathL (data not shown). Similarly, the Keratinase gene was removed from a cloning vector by digestion with restriction enzymes and cloned into the transfer vector pSynXIVVI+X3 generating the plasmid pSynKerat (data not shown). The recombinant viruses were constructed by separetely co-tranfecting insect cells with the pSynScathL and pSynKerat DNA and DNA from the recombinant vSynVI-gal in BTI-Tn5B1-4 cells. Within the insect cells, homologous recombination occurred between regions of the plasmid vector and viral genome. The recombinant viruses vSynScathL and vSynKerat were then isolated from the supernatant of co-transfected insect cells by end-point dilution (Figure 1).

### Bioassays

**Figure 2:**
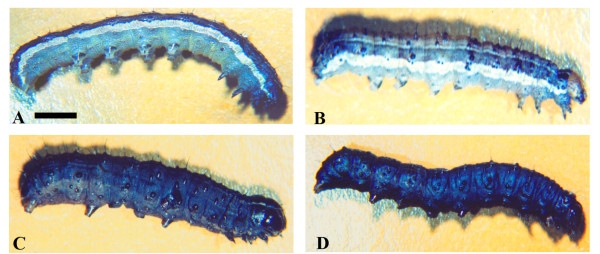
**Structural analysis of the cuticle of larvae of *S. frugiperda *observed in a stereomicroscope**. Uninfected larvae (A) and infected with type virus AcMNPV (120 h.p.i.) (B), recombinant vSynScathL (96 h.p.i.) (C), vSynKerat (96 h.p.i.) (D). Note melanization of cuticle in the larvae infected with vSynScathL and vSynKerat. Bar = 0.38 cm.

Thirty 3^rd ^instar *S. frugiperda *larvae were separetely inoculated with aproximately 10^6 ^pfu per larvae of each recombinant and wild type virus via hemolymph. The recombinants vSynScathL and vSynKerat were able to induce insect death faster than the wild-type virus (Table 1). The vSynScathL showed a LT_50 _and a mean time to death (TD) of 47 h and 2.62 days, respectively, while the AcMNPV, a LT_50 _of 136 h and a TD of 5.37 days, respectively. This represents a significant 65.5% reduction in the time needed to kill the virus infected insects when compared to the wild type virus. The LT_50 _and TD for the vSynKerat were 91 h and 3.70 days, respectively, with represents a reduction of 32.8% compared to AcMNPV. Moreover, in the final stages of infection, viruses with the ScathL and Kerat genes induced melanization of the cuticle, which was not observed with AcMNPV infected insects (Figure 2).

**Table 1:** LT_50 _values for the wild type and recombinant viruses in 3^rd ^instar *S. frugiperda *larvae.

Virus	**LT**_**50**_	CL (95%) Lower	CL (95%) Upper	TD/SD
**AcMNPV**	136.15	119.91	161.86	5.23(+/- 0.28)
**vSynScathL**	47.00	33.51	57.60	2.61(+/- 0.07)
**vSynKerat**	91.44	78.28	105.14	3.65(+/- 0.36)

LT_50_: Letal Time in 50% of the larvae, in hours

CL: conficdence limits at 95%

TD: mean time to death in days

SD: standard deviation

Larvae were injected with 10^6 ^pfu/larva into the hemolymph with the recombinant baculoviruses vSynScathL and vSynKerat and the wild type virus.

Droplet feeding bioassays were also carried out with neonate *S. frugiperda *larvae with different concentrations of occlusion bodies from AcMNPV, vSynScathL and vSynKerat. The recombinant vSynScathL was also shown to induce death in neonate larvae faster compared to wild-type virus (Table 2). The vSynScathL showed a LT_50 _of 77 h while the AcMNPV, a LT_50 _of 104 h when inoculated with 10^2 ^PIBs/nL. This represents a reduction of 26% in the time needed to kill the infected insects when compared to the wild type virus. The LT_50 _for the virus vSynKerat was 54 h, with a reduction of 48% compared to the virus AcMNPV. We also analysed the LC_50 _for the two recombinants but no significant diffference was observed when compared with the wild type virus (Table 3).

**Table 2:** LT_50 _values for the wild type and recombinants vSynScathL and vSynKerat in neonate *S. frugiperda *larvae.

Virus	**LT**_**50**_	CL (95%) lower	CL (95%) Upper	TD/SD
**AcMNPV**	104	94.07	112.05	4.16/(+/- 0.6)
**vSynScathL**	77	49.26	93.91	3.46/(+/-0.4)
**vSynKerat**	54	37.71	71.29	3.87(+/- 0.58)

LT_50_: Letal Time in 50% of the larvae, in hours

CL: conficdence limits at 95% TD: mean time to death in days

SD: standard deviation

Larvae were inoculated with 10^2 ^occlusion bodies/nL with the recombinant baculoviruses vSynScathL and vSynKerat and the wild type virus.

**Table 3:** LC_50 _values for the wild type and recombinants vSynScathL and vSynKerat in neonate *S. frugiperda *larvae.

Virus	**LC**_**50 **_ (occlusion bodies/nL)	CL (95%) Lower	CL (95%) Upper	*χ*^2 ^(df)
**AcMNPV**	32.32	19.10	47.84	1.49(3)
**vSynScathL**	8.15	1.73	26.72	3.99(3)
**vSynKerat**	30.41	17.58	51.17	1.28(3)

LC^50^: Letal Concentration in 50% of the larvae, in occlusion bodies/nL

CL: conficdence limits at 95%,

***χ*^2^**: qui-square

df: degrees of freedom.

Larvae were inoculated with different doses of occlusion bodies with the recombinant baculoviruses vSynScathL and vSynKerat and the wild type virus.

### Structural and ultrastructural tissue analysis of *S. frugiperda *larvae infectecd with different viruses

**Figure 3:**
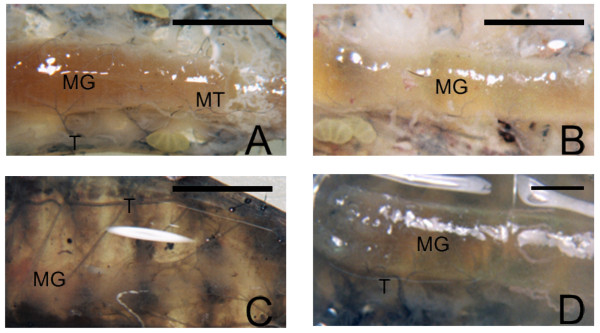
**Structural analysis of the internal tissues of larvae of *S. frugiperda***. Uninfected larvae (132 h.p.i) (A), infected larvae with the baculovirus AcMNPV (B), vSynScathL (C) and vSynKerat (D). In C and D in addition to the observed melanization of the cuticle, it is also possible to see the reduction of fat tissue.

**Figure 4:**
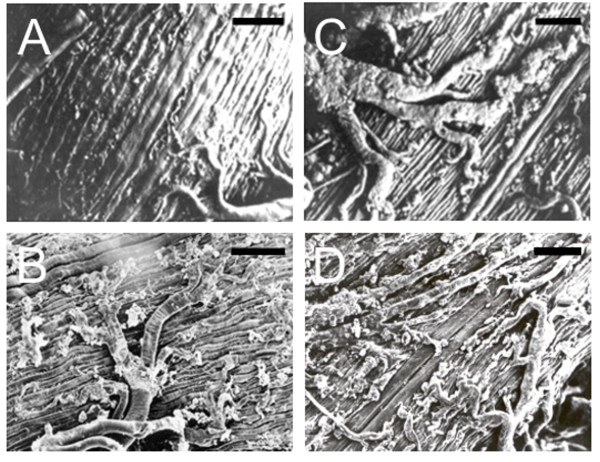
**Ultrastructure of *S. frugiperda *midgut from virus-infected insects at 96**. **h.p.i**. Scanning electron micrographs showing the integrity of the tissue around the gut of the caterpillar uninfected (A), tracheal system tightly attached to the midgut and partial destruction of the connective tissue in larvae infected with virus AcMNPV (B) and loosening of the tracheal system and intense tissue destruction in larvae infected with vSynScathL (C) and vSynKerat (D). Bar 100 μM.

*S. frugiperda *larvae uninfected and infected with AcMNPV, vSynScathL and vSynKerat were examined under a stereomicroscope (Figure 3) and a scanning electron microscope (Figure 4). The larvae infected with AcMNPV showed the presence of fat tissue (Figure 3) and tracheal system firmly attached to the gut of the caterpillar (Figure 4). On the other hand, larvae infected with vSynScathL (Figure 2) and vSynKerat (Figure 2) showed melanization of the cuticle, had little or no fat tissue and tracheal system was loosely connected to the midgut of the insect (Figure 4).

### Phenoloxidase activity

**Figure 5:**
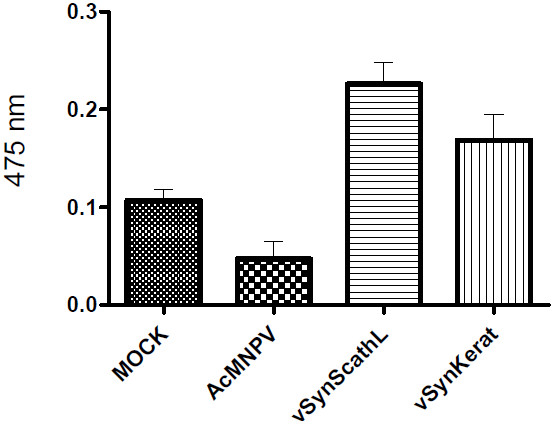
**Phenoloxidase activity in haemolymph of infected *S. frugiperda *larvae**. The haemolymph was collected at 72 h p.i., the cell removed by centrifugation and phenoloxidase activity was determined spectrophotometrically using the cell-free hemolymph (113 μg) and L-3, 4-dihydroxyphenylalanine (L-DOPA) as a substrate. The experiment was repeated 3 times. Note that haemolymph from insects infected with the recombinant vSynScathL and vSynKerat showed increased activation of the enzyme.

Phenoloxidase activity was determined spectrophotometrically by measuring formation of dopachrome from L-DOPA at 475 nm in haemolymph samples from insects infected with vSynScathL, vSynKerat, AcMNPV and mock infected (figure 5). We observed an expressive increase in phenoloxidase activity in haemolymph from *S. frugiperda *larvae infected with vSynScathL (0.23) and vSynKerat (0.17) when compared with haemolymph from mock-infected (0.10) and AcMNPV-infected insects (0.05). The experiment was repeated three times.

## Discussion

The introduction of heterologous genes into baculoviruses genomes has been performed for various purposes, such as to increase the virulence of these viruses towards their hosts [3,55] and for expression of heterologous proteins in cultured insect cells and insects [56,51,58].

Different genes have been introduced into the genome of baculovirus aiming the improvement of their pathogenicity towards their hosts. For instance, AcMNPV recombinants expressing wild type and mutated versions of JHE were able to improve viral pathogenicity and reduce the consumption of food by the larvae of *H. virescens *and *T. ni *[16,59,20]. The TxP-1 toxin gene from the mite *Pyemotes tritici*, was introduced into the genome of the AcMNPV and shown to have an improved insecticidal activity. The recombinant baculovirus expressing TxP-1 had a reduction of 30-40% in the time to induce insect death when compared to the wild type virus [60,13,61]. Similar results were found with the introduction of the scorpion toxin AaIT gene from *Androctonus australis *with lethal time reduced by 25-40% when compared to wild-type virus [11,12,62,8]. Other toxins from scorpions [63,64], spiders [65], sea anemones [65] and *B. thuringiensis *[34,35,37] were also expressed using recombinant baculoviruses, and most of them showed an improvement on the virus speed of kill. Strong promoters as those in the transfer vector pSynXIVVI+X3 [49,51] are widely used for high levels of heterologous protein expression in insect cells. This vector has two promoters in tanden (pSyn and PXIV) that are active from the viral late through the very late phases of transcription [49] and are responsible for the high levels of heterologous protein expression during infection. This vector also have the *polh *gene that facilitates detection and isolation of recombinant viruses when co-transfected with occlusion negative (occ^-^) viral DNA.

Recombinant baculoviruses expressing proteases that potentially degrade the basement membrane of tissues of insects have also been developed. A recombinant AcMNPV was constructed with the introduction of the ScathL gene from *S. peregrina*, under the command of the *p6.9 *promoter, and significantly reduced (49%) the survival time of infected neonate *H*. virescens larvae and the their consumption of food when compared to the wild type virus [38].

In this work, we inserted the genes of ScathL of *S. peregrina *and Keratinase of *A. fumigatus *in the genome of the baculovirus AcMNPV by using the vector pSynXIVVI+X3 and analysed the effect on viral pathogenicity. The recombinant vSynScathL constructed in this work confirmed the data previously shown by Harrison et al. [38] showing that the expression of the ScathL gene increase viral speed of kill when compared to the wild type AcMNPV. The recombinant vSynScathL showed a LT_50 _of 47 h while the AcMNPV, a LT_50 _of 136 h, which represents a significant reduction of 65.5% in the survival time of *S. frugiperda *when 10^6 ^pfu of BVs were innoculated into the hemolymph of third-instar larvae. Furhthermore, the vSynScathL showed a 26% reduction in survival time when neonate *S. frugiperda *larvae were orally inoculated with 10^2 ^occlusion bodies/nL. Harrison et al. [38] showed a 49% reduction in survival time of neonate *H. virescens *when infected with a AcMNPV recombinant containing the ScathL gene under the control of the *p6.9 *promoter (AcMLF9.ScathL) when compared to the wild type AcMNPV. Furthermore, Li et al. [66] have shown that purified ScathL was able to kill insects in the absence of baculovirus infection by injecting the protease into the hemocoel. The difference in larval survival time from the work by Harrison et al. [38] and this work, might be due to the diferent promoters used for the expression of the ScathL gene and the different viral susceptibilty of the insects tested, since *S. frugiperda *has been shown to be 1000 × less susceptible to AcMNPV by oral inoculaton when compared to the more susceptible *T. ni *larvae [67].

We also introduced the Keratinase (a serine protease) gene from the fungus *A. fumigatus *into the AcMNPV genome using the same vector and also showed an increase in viral speed of kill towards *S. frugiperda*. The virus vSynKerat showed a 32.8% reduction in the LT_50 _when compared to wild type virus when 10^6 ^pfu of BVs were innoculated into the hemolymph of third instar larvae and 48% reduction when 10^2 ^occlusion bodies/nL were administered to neonate larvae. Fungal serine proteases are known for their elastinolytic properties that enhance fungus invasiveness [68,69]. The production of *A. fumigatus *serine proteases capable of degrading elastin and mucin, among various other substrates has been previously observed [70]. Since the recombinant virus constructed in this work (vSynKerat) possesses a serine protease from *A. fumigatus *we would expect that the expression of this protein inside infected insect larvae would increase virus pathogenicity similarly to the ScathL by degrading extracellular matrix proteins and/or interfering with the phenoloxidase activity of the insect host. The LC_50 _for the two recombinants did not show significant diffferences when compared with the wild type virus (Table 2).

The melanization of the cuticle observed in insects infected with the recombinants vSynScathL and vSynKerat may have been caused by the activation of the insect phenoloxidase enzyme, found in the form of a pro-enzyme in the hemolymph. In invertebrates, the presence of antigens and the appearance of tissue damage results in the deposition of melanin around the damaged tissue or antigen as well as sclerotization of the cuticle [71]. Melanization of the cuticle and tissue damage, including rupture of the intestine and fragmentation of the fat tissue has been previously shown in larvae of *H. virescens *infected with a recombinant AcMNPV containing the ScathL gene [38,72,73], suggesting that ScathL was able to cause tissue fragmentation prior to insect death and activate the cascade triggered by serine proteases leading to conversion of pro-phenoloxidase in its active form phenoloxidase. However, Li et al. [66] have shown that the cystein protease activity of purified ScathL was not able to activate pro-phenoloxidase to phenoloxidase *in vitro *and the phenoloxidase activity in the hemolymph of *H. virescens *larvae was not altered by a recombinant AcMNPV containing the ScathL gene under the baculovirus basic *p6.9 *promoter (AcMLF9.ScathL).

We have shown that both recombinants (vSynScathL and vSynKerat) containing the ScathL and Keratinase genes under the command of strong promoters were able to increase phenoloxidase activity in the hemolymph of *S. frugiperda *larva. Since the Keratinase is a serine protease this result was not a surprise, since insect serine proteases are known to be involved in melanin production [71]. The increased hemolymph phenoloxidase activity by the vSynScathL could be explained, in part, by the high level of expression of this protein in infected insects. However, further analysis will be necessary to clarify the role of the ScathL in this increase in pheoloxidase activity.

## Conclusions

Although recombinant baculoviruses have not yet been widely used for the control of insect pests, they constitute a viable alternative to chemical insecticides. The recombinant baculoviruses containing protease genes can be added to list of engineered baculoviruses with great potential to be used in integrated pest management programs.

## Competing interests

The authors declare that they have no competing interests.

## Authors' contributions

AWG carried out the study, performed analysis of data and drafted the manuscript SP helped with the construction of recombinant viruses and with the structural and ultrastructural analysis of virus-infected *S. frugiperda *larvae. RLBS helped with bioassays. EFN and CRF developed the phenoloxidase assay protocol and provided the Keratinase gene. TN participated in the study design and sequencing of DNA constructs. BMR conceived the study, provided research funds, students supervision and revised the manuscript. All authors have read and approved the final manuscript.
